# SOCIOLOGY AND GERONTOLOGY: NEW PERSPECTIVES AND VITAL ISSUES

**DOI:** 10.1093/geroni/igae098.1969

**Published:** 2024-12-31

**Authors:** Stephen Katz

**Affiliations:** Chair

## Abstract

The relationship between sociology and gerontology continues to forge dynamic pathways that cross critical thinking with innovative methodologies and radical advocacy. Sociological studies (and its specialties) have deepened understanding of aging by exploring its social contexts, life-course trajectories, intersecting inequalities, lifestyle cultures, social determinants of health, and political governance. Despite such advances, mainstream research is criticized for resisting their implications by returning ‘to the safety of traditional, individual-level explanations’ (Dannefer 2022). This trend parallels an increasing neglect of the social dimension of aging in technocratic policy-making. This symposium addresses these challenges by presenting research on the vital and everyday issues of aging, both to strengthen the sociological lens in gerontology and to explore new perspectives on social changes affecting aging societies. Stephen Katz reviews the loneliness ‘epidemic’ amongst older adults and the limitations of diagnostic approaches, suggesting instead how historical and qualitative research can formulate loneliness within diversely lived social and material environments. Chris Phillipson examines the sociological consequences of COVID-19 and its impact and consequences for different groups of older people, and discusses the role of social science in contributing to policy responses to the pandemic. Vera Gallistl and Anna Wanka explain the importance of materialist sociological theories in expanding aging relationships to include the interplay of human, more-than-human and non-human actors in digital societies. Amanda Grenier provides a critical overview of the disjuncture between professional knowledge-production and aging experiences, offering a radical proposal for an ethnographic case-study methodology to tackle issues of frailty and mobility.

